# Supplementary material for: An evidence synthesis approach for combining different data sources illustrated using entomological efficacy of insecticides for indoor residual spraying
Source: PLoS One. 2022 Mar 24;17(3):e0263446. doi: 10.1371/journal.pone.0263446 (PMC8947499; doi:10.1371/journal.pone.0263446)
Supplement: S2 Appendix — (PDF) [file pone.0263446.s002.pdf]

An evidence synthesis approach for combining different data sources illustrated using entomological efficacy of insecticides for indoor residual spraying

Nathan Green* Fiacre Agossa Boulais Yovogan Richard Oxborough
Jovin Kitau Pie Muller Edi Constant Mark Rowland Emile FS Tchacaya
Koudou G Benjamin Thomas S Churcher Ellie Sherrard-Smith

2021-12-13

Supplementary Material 1

We built statistical models coded in OpenBUGS Release 3.2.3 [1] and Stan [2] and fitted using a Bayesian framework.

The first model assesses the aggregated data only and estimates the proportion of mosquitoes that are killed and those that are blood-fed before inferring from the product of these probabilities, those that are surviving and blood-feeding, assumption that a mosquito is equally likely to be blood-fed whether killed or not.

The second model adjusts the aggregated data directly prior to fitting the model and then fits a logistic binomial to estimate the respective probabilities from the adjusted data. This is the approach used in Sherrard-Smith *et al* (2018)[3] on the aggregated data. This model structure is also fitted to the comprehensive data where no prior assumptions are required (Figure 2c) to estimate the probabilities from the comprehensive data set.

OpenBUGS code

```
## using 4 category data only ('comprehensive model')

model {

  ## separate binomial models
  ## for successfully fed and dead

  for(j in 1:len_b){

      X_sf[j] ~ dbin(prob_sf[j], Nb[j])
      logit(prob_sf[j]) <- beta0[studyid_b[j]] + beta1[studyid_b[j]]*time_b[j]

      X_d[j] ~ dbin(prob_d[j], Nb[j])
      logit(prob_d[j]) <- beta0d[studyid_b[j]] + beta1d[studyid_b[j]]*time_b[j]
    }

  ## global level
```

*Department of Statistical Science, University College London, Gower Street, London WC1E 6BT, UK, n.green@ucl.ac.uk

```
  for (i in 1:N_studies_b){

    beta0[i] ~ dnorm(mu_beta0, tau_beta0)
    beta1[i] ~ dnorm(mu_beta1, tau_beta1)

    beta0d[i] ~ dnorm(mu_beta0d, tau_beta0d)
    beta1d[i] ~ dnorm(mu_beta1d, tau_beta1d)
  }

  ## node transformations

  tau_beta1 <- 1/sigma2_beta1                   # precision for between trials
  sigma_beta1 <- exp(logsigma_beta1)    # sd for between trials
  sigma2_beta1 <- pow(sigma_beta1, 2)   # variance for between trials

  tau_beta0 <- 1/sigma2_beta0                   # precision for between trials
  sigma_beta0 <- exp(logsigma_beta0)    # sd for between trials
  sigma2_beta0 <- pow(sigma_beta0, 2)   # variance for between trials

  tau_beta1d <- 1/sigma2_beta1d                 # precision for between trials
  sigma_beta1d <- exp(logsigma_beta1d)  # sd for between trials
  sigma2_beta1d <- pow(sigma_beta1d, 2) # variance for between trials

  tau_beta0d <- 1/sigma2_beta0d                 # precision for between trials
  sigma_beta0d <- exp(logsigma_beta0d)  # sd for between trials
  sigma2_beta0d <- pow(sigma_beta0d, 2) # variance for between trials

  ## prior distributions

  mu_beta0 ~ dnorm(0, 1.0E-6)
  logsigma_beta0 ~ dunif(-5, 10)              # log-sd for between trials
  mu_beta1 ~ dnorm(0, 1.0E-6)
  logsigma_beta1 ~ dunif(-5, 10)              # log-sd for between trials

  mu_beta0d ~ dnorm(0, 1.0E-6)
  logsigma_beta0d ~ dunif(-5, 10)             # log-sd for between trials
  mu_beta1d ~ dnorm(0, 1.0E-6)
  logsigma_beta1d ~ dunif(-5, 10)             # log-sd for between trials

  for (t in 1:12) {

    ## posterior predictions

    logit(pred_sf[t]) <- mu_beta0 + mu_beta1*t
    logit(pred_d[t]) <- mu_beta0d + mu_beta1d*t

    for (j in 1:N_studies_b) {

        logit(predj_sf[j,t]) <- beta0[j] + beta1[j]*t
        logit(predj_d[j,t]) <- beta0d[j] + beta1d[j]*t
    }
  }
}
```

The third model, a Bayesian evidence synthesis model, combines the data resources probabilistically to incorporate the inferences that can be made from the comprehensive data benefited by the additional aggregated data resource.

```
## full evidence synthesis model

model {

  ## 2 groups ##
  ## binomial models

  for(j in 1:len_a){

      Xd[j] ~ dbin(prob_a[j, 2] + prob_a[j, 4], Na[j])
      Xf[j] ~ dbin(prob_a[j, 1] + prob_a[j, 2], Na[j])

      phi_a[j,1] <- 1
      prob_a[j,1] <- 1/sum(phi_a[j, 1:4])

      for(c in 2:4){

        log(phi_a[j,c]) <- beta0c[1, studyid_a[j], c] + beta1c[1, studyid_a[j], c]*time_a[j]
        prob_a[j,c] <- phi_a[j,c]/sum(phi_a[j, 1:4])
      }
    }

  ## 4 groups ##
  ## multinomial model

  for(j in 1:len_b){

      X_b[j, 1:4] ~ dmulti(prob_b[j, 1:4], Nb[j])

      phi_b[j,1] <- 1
      prob_b[j,1] <- 1/sum(phi_b[j, 1:4])

      for(c in 2:4){

        log(phi_b[j,c]) <- beta0c[2, studyid_b[j], c] + beta1c[2, studyid_b[j], c]*time_b[j]
        prob_b[j,c] <- phi_b[j,c]/sum(phi_b[j, 1:4])
      }
    }

  ## global level

  for (i in 1:N_studies_a){

    #set reference category to zero
    beta0c[1,i,1] <- 0
    beta1c[1,i,1] <- 0

    for(c in 2:4){

      beta0c[1,i,c] ~ dnorm(mu_beta0[c], tau_beta0[c])
```

```
    beta1c[1,i,c] ~ dnorm(mu_beta1[c], tau_beta1[c])
  }
}

for (i in 1:N_studies_b){

  #set reference category to zero
  beta0c[2,i,1] <- 0
  beta1c[2,i,1] <- 0

  for(c in 2:4){

    beta0c[2,i,c] ~ dnorm(mu_beta0[c], tau_beta0[c])
    beta1c[2,i,c] ~ dnorm(mu_beta1[c], tau_beta1[c])
  }
}

## node transformations

for(c in 2:4){

  tau_beta1[c] <- 1/sigma2_beta1[c]                   # precision for between trials
  sigma_beta1[c] <- exp(logsigma_beta1[c])    # sd for between trials
  sigma2_beta1[c] <- pow(sigma_beta1[c], 2)   # variance for between trials

  tau_beta0[c] <- 1/sigma2_beta0[c]                   # precision for between trials
  sigma_beta0[c] <- exp(logsigma_beta0[c])    # sd for between trials
  sigma2_beta0[c] <- pow(sigma_beta0[c], 2)   # variance for between trials
}

## prior distributions

for(c in 2:4){

  mu_beta0[c] ~ dnorm(0, 1.0E-6)
  logsigma_beta0[c] ~ dunif(-5, 10)           # log-sd for between trials
  mu_beta1[c] ~ dnorm(0, 1.0E-6)
  logsigma_beta1[c] ~ dunif(-5, 10)           # log-sd for between trials
}

for (t in 1:12) {

  ## posterior predictions

  phi_pred[t,1] <- 1
  prob_pred[t,1] <- 1/sum(phi_pred[t, 1:4])

  for(c in 2:4){

    log(phi_pred[t,c]) <- mu_beta0[c] + mu_beta1[c]*t
    prob_pred[t,c] <- phi_pred[t,c]/sum(phi_pred[t, 1:4])
  }
```

```
    pred_d[t] <- prob_pred[t,2] + prob_pred[t,4]
    pred_f[t] <- prob_pred[t,1] + prob_pred[t,2]
    pred_sf[t] <- prob_pred[t,1]

    # (df/dn) / (sf/sn)
    OR[t] <- (prob_pred[t,2]/prob_pred[t,4])/(prob_pred[t,1]/prob_pred[t,3])
  }
}
```

The following script can be used to run these models.

```
library(R2jags)
library(R2WinBUGS)
library(purrr)

data_a <- read.csv(here::here("code", "data input", "N2_data.csv"), header = TRUE)
data_b <- read.csv(here::here("code", "data input", "N4_data.csv"), header = TRUE)

jags_dat_input <-
  list(
    ## a
    len_a = nrow(data_a), #number of data points of type a
    Na = data_a$N_total, #total number of mosquitos in each trial type a
    Xd = data_a$N_dead,
    Xf = data_a$N_fed,
    time_a = data_a$months_since_IRS,
    N_studies_a = length(unique(data_a$study_id)),
    studyid_a = data_a$study_id,
    ## b
    len_b = nrow(data_b), #number of data points of type b
    Nb = data_b$N_total,  #total number of mosquitos in each trial type b
    time_b = data_b$months_since_IRS,
    N_studies_b = length(unique(data_b$study_id)),
    studyid_b = data_b$study_id,
    X_b = with(data_b,
               cbind(N_survived_fed = Nsf + Nsfe,
                     N_dead_fed = Ndf + Ndfe,
                     N_survived_unfed = Nsn + Nsne,
                     N_dead_unfed = Ndn + Ndne))
  )


params <-
  c("mu_beta0", "sigma_beta0",
    "mu_beta1", "sigma_beta1",
    "pred_d", "pred_sf",
    "pred_f",
    "OR"
  )

##############
## run MCMC ##
##############
```

```
out <- jags(jags_dat_input,
            # inits = list(inits(), inits()),
            parameters.to.save = params,
            model.file = here::here("code", "BUGS_code_evidsynth.txt"),
            n.chains = 2,
            n.iter = n_iter,
            n.burnin = n_burnin,
            n.thin = n_thin,
            DIC = TRUE,
            working.directory = here::here("code"),
            progress.bar = "text")

BUGSoutput <- out$BUGSoutput

save(BUGSoutput, file = here::here("code", "data output", "BUGSoutput_evidsynth.RData"))
```

Stan code

For data point i, study k and group $j = 2, 3, 4$,

$$\beta_k^j = \mu_\beta^j + \tau_{\beta^j}\tilde{\beta}_k^j$$
$$\alpha_k^j = \mu_\alpha^j + \tau_{\alpha^j}\tilde{\alpha}_k^j$$

$$P_{k,i}^1 = \frac{1}{1 + \sum_{l=2}^4 \exp(-(\alpha_k^l + \beta_k^l \times t_{k,i}^l))}$$
$$P_{k,i}^j = \frac{\exp(-(\alpha_k^j + \beta_k^j \times t_{k,i}^j))}{1 + \sum_{l=2}^4 \exp(-(\alpha_k^l + \beta_k^l \times t_{k,i}^l))}$$

The for the comprehensive data

$$X_{k,i}\ Multinomial(N_{k,i}, P_{k,i})$$

And for the aggregate data

$$X_{k,i}^d\ Binomial(N_{k,i}, P_{k,i}^{df} + P_{k,i}^{df})$$
$$X_{k,i}^f\ Binomial(N_{k,i}, P_{k,i}^{df} + P_{k,i}^{df})$$

```
data {
  int<lower=1> N_groups; // Number of reponse categories

  int<lower=1> N_agg_exp;           // Number of experiments with aggregated responses
  int<lower=0> k1_agg[N_agg_exp];   // Number of successes in first aggregation pattern
  int<lower=0> k2_agg[N_agg_exp];   // Number of successes in second aggregation pattern
  int<lower=1> N_trials_agg[N_agg_exp]; // Number of trials

  int<lower=1> N_studies;
  int<lower=1, upper=N_studies> study_idx_agg[N_agg_exp]; // Study index

  real time_agg[N_agg_exp];

  int<lower=1> N_indiv_exp;   // Number of experiments with individual responses
  int<lower=0> N_responses_indiv[N_indiv_exp, N_groups]; // Counts of each response
```

```
  int<lower=1, upper=N_studies> study_idx_indiv[N_indiv_exp]; // Study index

  real time_indiv[N_indiv_exp];
}

parameters {
  real mu_alpha[N_groups];           // Intercept population location
  real<lower=0> tau_alpha;           // Intercept population scale

  real alpha_tilde[N_studies, N_groups - 1]; // Noncentered intercepts

  real mu_beta[N_groups];           // Slope population location
  real<lower=0> tau_beta;           // Slope population scale

  real beta_tilde[N_studies, N_groups - 1]; // Noncentered slopes
}

model {
  // priors
  //for (i in 1:N_groups)
    mu_alpha ~ normal(0, 2);

  tau_alpha ~ normal(0, 2);
  //to_vector(alpha_tilde) ~ normal(0, 1); //this should be a better for()
  for (n in 1:N_studies)
    alpha_tilde[n] ~ normal(0, 1);

  //for (i in 1:(N_groups - 1))
    mu_beta ~ normal(0, 0.4);

  tau_beta ~ normal(0, 0.4);

  for (n in 1:N_studies)
    beta_tilde[n] ~ normal(0, 1);

  // aggregate
  for (n in 1:N_agg_exp) {

    //real eta[N_groups]; // Latent effect for each response
    vector[N_groups] eta;

    //real p[N_groups];   // Response probabilities
    vector[N_groups] p;

    eta[1] = 0;
    for (g in 2:N_groups) {

      real alpha = mu_alpha[g] + tau_alpha * alpha_tilde[study_idx_agg[n], g - 1];
      real beta = mu_beta[g] + tau_beta * beta_tilde[study_idx_agg[n], g - 1];
      eta[g] = alpha + beta * time_agg[n];
    }

    p = softmax(eta);
```

```
    k1_agg[n] ~ binomial(N_trials_agg[n], p[2] + p[4]);
    k2_agg[n] ~ binomial(N_trials_agg[n], p[1] + p[2]);
  }

  // individual
  for (n in 1:N_indiv_exp) {

    //real eta[N_groups]; // Latent effect for each response
    vector[N_groups] eta;

    //real p[N_groups];   // Response probabilities
    vector[N_groups] p;

    eta[1] = 0;
    for (g in 2:N_groups) {
      real alpha = mu_alpha[g] + tau_alpha * alpha_tilde[study_idx_indiv[n], g - 1];
      real beta = mu_beta[g] + tau_beta * beta_tilde[study_idx_indiv[n], g - 1];
      eta[g] = alpha + beta * time_indiv[n];
    }

    p = softmax(eta);

    N_responses_indiv[n] ~ multinomial(p);
  }
}

generated quantities {

  matrix[N_groups, 12] p_pred;

  for (n in 1:12) {
    vector[N_groups] eta;

    eta[1] = 0;
    for (g in 2:N_groups) {

      eta[g] = mu_alpha[g] + mu_beta[g] * n;
    }

    p_pred[, n] = softmax(eta);
  }

}
```

References

1. Lunn DJ, Thomas A, Best N, Spiegelhalter DJ. WinBUGS – a Bayesian modelling framework: concepts, structure, and extensibility. Statistics and Computing. 2000;10: 325–337.
2. Stan Development Team. Stan Modeling Language: User's Guide and Reference Manual. 2017 pp. 1–488.
3. Sherrard-Smith E, Griffin JT, Winskill P, Corbel V, Pennetier C, Djénontin A, et al. Systematic review of indoor residual spray efficacy and effectiveness against Plasmodium falciparum in Africa. Nature Communications. 2018;9: 4982. doi:10.1038/s41467-018-07357-w
